# The diagnosis of intramural esophageal squamous cell carcinoma without mucosal invasion using endoscopic ultrasound-guided fine needle aspiration biopsy: A case report

**DOI:** 10.1097/MD.0000000000021099

**Published:** 2020-07-02

**Authors:** Hanghai Pan, XinXin Zhou, Fei Zhao, Guochun Lou

**Affiliations:** a Department of Gastroenterology, Zhejiang Provincial People's Hospital, People's Hospital of Hangzhou Medical College; b Department of Gastroenterology, The First Affiliated Hospital, College of Medicine, Zhejiang University, Zhejiang Province, China.

**Keywords:** endoscopic mucosal biopsy, EUS-FNA, intramural esophageal squamous cell carcinoma

## Abstract

**Rationale:**

Intramural esophageal squamous cell carcinoma (ESCC) without mucosal invasion is extremely rare. Endoscopic mucosal biopsy results are often negative, making diagnosis difficult. In these cases, endoscopic ultrasound-guided fine needle aspiration (EUS-FNA) biopsy is a useful diagnostic method.

**Patient concerns:**

A 78-year-old female was admitted to hospital due to dysphagia, and gastroscopy showed a concentric narrowing of the esophageal lumen with a smooth and undamaged esophageal mucosa.

**Diagnoses:**

Endoscopic ultrasound (EUS) revealed that the esophageal mucosa was thickened with a low echo, and the layers of the esophageal wall could not be clearly distinguished. Cytologic and pathologic diagnoses were obtained through EUS-FNA, which suggested ESCC.

**Interventions:**

According to the pathologic diagnosis obtained by EUS-FNA, surgery or radiotherapy were recommended for this patient. Eventually, this patient elected to seek treatment at another medical institution.

**Outcomes:**

This type of disease cannot be diagnosed according to gastroscopic biopsy alone, and the diagnosis was eventually confirmed through EUS-FNA.

**Lessons:**

When an imaging examination suggests a possible malignant lesion of the oesophagus, EUS-FNA may be considered if the surface mucosa contains no endoscopic damage. EUS-FNA has high diagnostic value with high sensitivity, minimal invasiveness, and high safety.

## 1 Introduction

Esophageal cancer is one of the most common malignant tumors of the digestive tract and mainly includes esophageal squamous cell carcinoma (ESCC) and esophageal adenocarcinoma.^[1]^ ESCC accounts for approximately 88% of all esophageal cancers and an even higher proportion in Eastern countries.^[2]^ Progressive ESCC often presents with dysphagia and is endoscopically characterized by irregular bulging masses, ulcers, and luminal stenosis. Different degrees of esophageal mucosal invasion and damage are present in almost all esophageal cancers, and gastroscopy and pathological biopsy can usually confirm the diagnosis.^[3]^ In rare cases, ESCC does not appear to have invaded the mucosal surface, and thus far, only 5 cases have been reported in the literature.^[4–8]^ Gastroscopy often shows that this type of cancer has a smooth esophageal mucosa free of damage and that it often presents only as an esophageal submucosal bulge or esophageal narrowing with negative pathological biopsy results, which makes diagnosis very difficult and may even result in misdiagnosis. Here, we report a rare case of advanced intramural ESCC with concentric narrowing of the esophageal lumen and intact esophageal mucosa using endoscopic ultrasound-guided fine needle aspiration (EUS-FNA) biopsy. Endoscopic ultrasound (EUS) suggested that this esophageal cancer had invaded all layers of the wall, and EUS-FNA pathology confirmed the diagnosis of ESCC.

## 2 Case presentation

**Figure 1:**
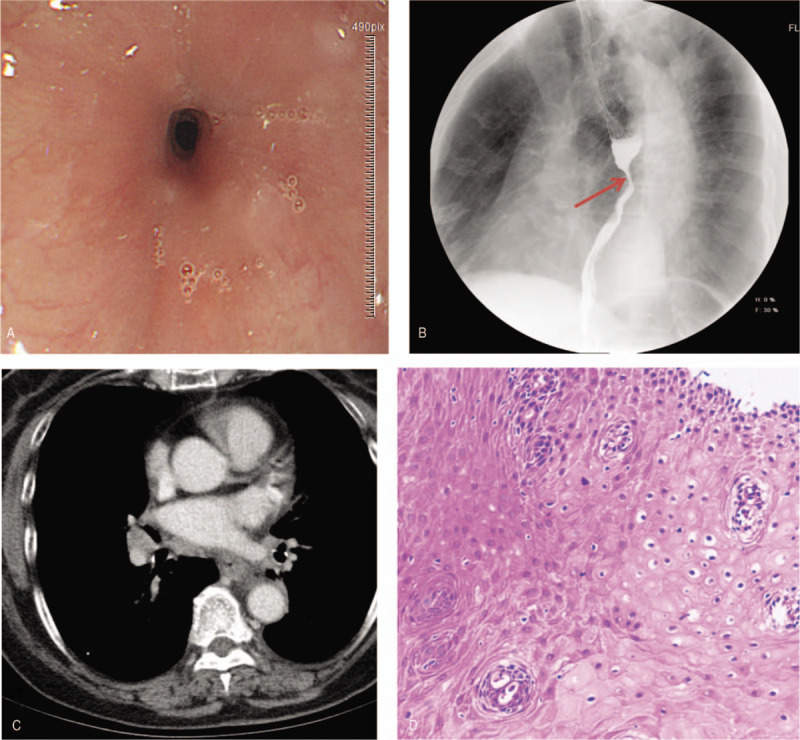
(A) Gastroscopy demonstrated concentric narrowing of the esophageal lumen at 30 cm from the central inciso. The mucosa overlying the stricture was smooth.(B) Barium esophagography showed a narrowing of approximately 1.5 cm in length in the middle and lower esophagus without visible disruption of the mucosa.(C) CT revealed that the wall of the middle and lower esophagus was obviously thickened.(D) The biopsy results suggested squamous epithelial hyperplasia.

A 78-year-old woman presented with dysphagia of solid food for 1 month, which was associated with occasional nausea and vomiting. The patient denied noticeable weight loss, hoarseness, caustic ingestion, and a history of tuberculosis and tumors, among other conditions. After admission, gastroscopy was performed, which demonstrated concentric narrowing of the esophageal lumen at 30 cm from the central incisor; the lumen was approximately 4 mm in size, and the gastroscope could not reach beyond this point. The mucosa overlying the stricture was smooth, and biopsy was performed at this site (Fig. 1A). Subsequently, the patient underwent barium esophagography and contrast-enhanced thoracic computed tomography (CT). Barium esophagography showed a narrowing of approximately 1.5 cm in length in the middle and lower esophagus without visible disruption of the mucosa; lumen expansion was limited, but the barium was still able to pass through the area (Fig. 1B). Contrast-enhanced thoracic CT revealed that the wall of the middle and lower oesophagus was obviously thickened, which indicated possible malignancy (Fig. 1C). However, the biopsy results suggested squamous epithelial hyperplasia (Fig. 1D).

**Figure 2:**
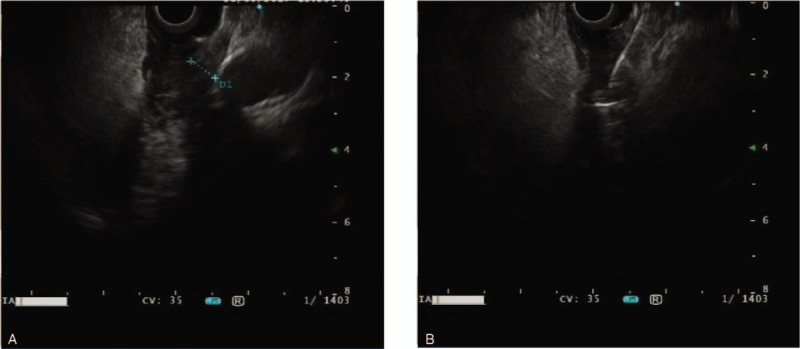
(A) EUS revealed that the mucosa overlying the esophageal lesion was significantly thickened and was approximately 8.7 mm thick in the thicker region, with a low echo; the layers of the esophageal wall could not be clearly recognized. (B) EUS-FNA was performed 3 times at the thickened esophageal wall.

**Figure 3:**
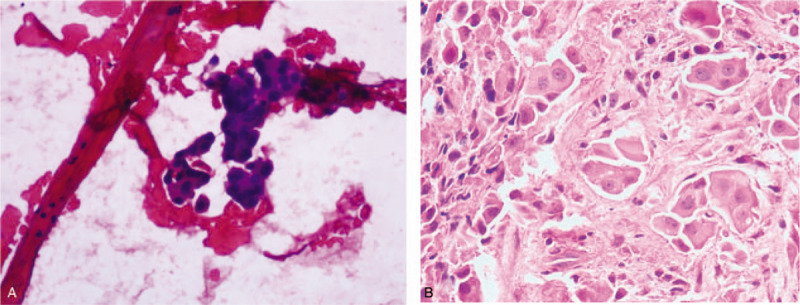
(A) Cancer cells were observed in the aspiration cytology specimen. (B) Histopathological haematoxylin and eosin staining revealed atypical epithelial cells.

**Figure 4:**
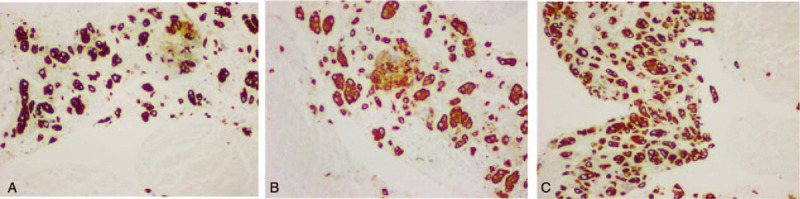
The immunohistochemistry results indicated cytokeratin CK34βE12 (+++), CK Pan (+++), CK5/6 (+++), which supported a diagnosis of SCC. (A) CK34βE12 (B) CK Pan (C) CK5/6.

For this patient, gastroscopy indicated only luminal stenosis without the presence of ulcers or masses in the mucosa, and the biopsy was negative for malignancy. However, CT examination revealed a significant thickening of the esophageal wall. It was necessary to determine a better method by which the esophageal lesions could be diagnosed in this patient. EUS revealed that the mucosa overlying the esophageal lesion was significantly thickened and was approximately 8.7 mm thick in the thicker region, with a low echo; the layers of the esophageal wall could not be clearly recognized (Fig. 2A). Then, in Doppler mode, we performed EUS-FNA 3 times at the thickened esophageal wall (Fig. 2B). The aspiration smear was sent for aspiration cytology, and the tissue strip was sent for pathological examination. Cancer cells were observed in the aspiration cytology specimen (Fig. 3A), and histopathological haematoxylin and eosin (H&E) staining revealed atypical epithelial cells, which indicated a high possibility of squamous cell carcinoma (SCC) (Fig. 3B). The immunohistochemistry results indicated cytokeratin CK34βE12 (+++), CK Pan (+++), CK5/6 (+++), and smooth muscle actin SMA(-), which supported a diagnosis of SCC (Fig. 4). Surgery or radiotherapy was recommended, but this patient eventually elected to seek treatment at another medical institution.

## 3 Discussion

ESCC is a malignant tumor that originates from esophageal squamous epithelial cells and has invasive growth. As such, almost all ESCCs, especially advanced ESCCs, invade mucosal membranes. Endoscopically, early ESCC is characterized by altered color, erosion, superficial depression, or elevation of the esophageal mucosa.^[9]^ Progressive ESCC usually presents as a bulging mass possibly accompanied by ulcers, which grows towards the esophageal lumen. It is easy to obtain a definite pathologic diagnosis through endoscopic biopsy. Oesophagography often shows the disruption of mucosal folds and damage.^[10]^

For this patient, although significant esophageal lumen narrowing was observed, the mucosa was smooth and did not appear damaged or exhibit a bulging mass, and no apparent interruption of the esophageal mucosal folds was seen on barium esophagography. Moreover, the mucosal biopsy was negative for malignancy, which made the diagnosis difficult. Very few cases with similarly unusual growth patterns, in which the growth was only on the inside of the wall with no invasion of the mucosal epithelium, have been previously reported. In these previous reports, this type of ESCC was termed intramural esophageal squamous cell carcinoma or esophageal squamous cell carcinoma with an entirely intramural growth pattern.^[6,8]^ Currently, the specific mechanism underlying this unusual growth pattern remains unclear. McGregor et al found cysts in the lesioned esophageal wall at autopsy. ESCC that did not invade the mucosal epithelium may have originated from the squamous cells of the epithelium of the submucosal cyst.^[4]^ However, Rahden et al did not find esophageal cysts in the lesions of other patients with a confirmed diagnosis by surgical pathology.^[6]^

Since endoscopic mucosal biopsy is often negative for malignancy in such cases, a confirmed pathologic diagnosis cannot be achieved until after surgery. Only 1 case has been clearly diagnosed through EUS-FNA.^[8]^ However, the final diagnosis was only based on cytology. In our case, we confirmed both the cytologic and pathologic diagnosis through EUS-FNA. No complications such as bleeding or infection occurred. In our experience, EUS-FNA is minimally invasive, safe, and has high diagnostic value for the diagnosis of intramural ESCC without mucosal invasion.

## 4 Conclusion

Here, we report an extremely rare type of ESCC in which tumor cells grow only in the esophageal wall and do not invade the esophageal mucosa. A pathologic diagnosis cannot be obtained through gastroscopy for this type of lesion, but EUS-FNA has high diagnostic value with minimal invasiveness and high safety.

## Author contributions

**Data curation:** Fei Zhao, Guochun Lou.

**Funding acquisition:** Hanghai Pan.

**Writing – original draft:** Hanghai Pan, XinXin Zhou

**Writing – review & editing:** Fei Zhao.
